# Role of sirtuins in bone biology: Potential implications for novel therapeutic strategies for osteoporosis

**DOI:** 10.1111/acel.13301

**Published:** 2021-01-04

**Authors:** Qiangqiang Li, Jack Chun‐yiu Cheng, Qing Jiang, Wayne Yuk‐wai Lee

**Affiliations:** ^1^ SH Ho Scoliosis Research Laboratory Department of Orthopaedics and Traumatology The Chinese University of Hong Kong Hong Kong SAR China; ^2^ Joint Scoliosis Research Center of the Chinese University of Hong Kong and Nanjing University The Chinese University of Hong Kong Hong Kong SAR China; ^3^ Li Ka Shing Institute of Health Sciences The Chinese University of Hong Kong Hong Kong SAR China; ^4^ Department of Sports Medicine and Adult Reconstructive Surgery Drum Tower Hospital affiliated to Medical School of Nanjing University Nanjing China

**Keywords:** aging, bone remodeling, osteoporosis, sirtuins

## Abstract

The decline in bone mass and bone strength and musculoskeletal problems associated with aging constitute a major challenge for affected individuals and the healthcare system globally. Sirtuins 1‐7 (SIRT1‐SIRT7) are a family of nicotinamide adenine dinucleotide‐dependent deacetylases with remarkable abilities to promote longevity and counteract age‐related diseases. Sirtuin knockout and transgenic models have provided novel insights into the function and signaling of these proteins in bone homeostasis. Studies have revealed that sirtuins play a critical role in normal skeletal development and homeostasis through their direct action on bone cells and that their dysregulation might contribute to different bone diseases. Preclinical studies have demonstrated that mice treated with sirtuin agonists show protection against age‐related, postmenopausal, and immobilization‐induced osteoporosis. These findings suggest that sirtuins could be potential targets for the modulation of the imbalance in bone remodeling and treatment of osteoporosis and other bone disorders. The aim of this review was to provide a comprehensive updated review of the current knowledge on sirtuin biology, focusing specifically on their roles in bone homeostasis and osteoporosis, and potential pharmacological interventions targeting sirtuins for the treatment of osteoporosis.

## Graphical abstract

Sirtuins (SIRT1–SIRT7) are a family of nicotinamide adenine dinucleotide (NAD+)‐dependent deacetylases with remarkable abilities to promote longevity and counteract age‐related diseases. Sirtuins play diverse roles in bone homeostasis through actions on various cell types in bone tissues. Preclinical and clinical evidence have demonstrated the link between sirtuins and osteoporosis. Modulation of sirtuins could be potential strategies for the treatment of osteoporosis and other aging‐related bone disorders.

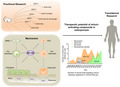

**Abbreviations**
ATP: adenosine triphosphate
BMD: bone mineral density
DSB: double‐strand break
H_2_O_2_: hydrogen peroxide
IL‐1: interleukin 1
IL‐6: interleukin 6
NHEJ: non‐homologous end joining
OA: osteoarthritis
OPG: osteoprotegerin
ROS: reactive oxygen species
SASP: senescence‐associated secretory phenotype
SNP: Single nucleotide polymorphism
SOD2: superoxide dismutase 2
TNF‐α: tumor necrosis factor alpha

## 1 INTRODUCTION

Bone is a dynamic tissue that is constantly adapting its structure to functional demands through processes tightly regulated by two principal cell types: osteoblasts and osteoclasts (Harada & Rodan, 2003). Imbalanced action of these two cell types disrupts bone formation and resorption, leading directly or indirectly to the manifestation of various bone diseases (Rodan & Martin, 2000). Osteoporosis is a major public health concern worldwide, particularly in aging societies (Gullberg et al., 1997; Zeng et al., 2019). Current pharmacological treatments of osteoporosis primarily aim either to reduce excessive osteoclastic bone resorption (e.g., estrogen and bisphosphonates) or to promote osteoblastic bone formation (e.g., parathyroid hormone), and to a lesser degree, to achieve both (e.g., anti‐sclerostin [SOST] antibody). The primary cause of this imbalance in bone cell activity observed with aging is not yet fully understood. In addition, there is growing concern about the long‐term use of these drugs due to their off‐target effects (Davis et al., 2016; Kim et al., 2016; Lv et al., 2020; Silverman & Landesberg, 2009). There is a clear demand for continuing efforts in the research and development of safer preventative and/or therapeutic agents. Mitochondrial dysfunction (McBride et al., 2006; Trifunovic & Larsson, 2008; Trifunovic et al., 2004; Varanasi et al., 1999), oxidative stress (Balaban et al., 2005; Cadenas & Davies, 2000), and inflammation (El Assar et al., 2013; Woods et al., 2012) are found to be closely related with the impairment of cellular homeostasis and contribute to the progression of age‐related diseases, including osteoporosis. Therefore, targeting these primary aging processes represents a new class of treatment strategies for multiple aging tissues, including bone.

Mammalian sirtuins play important roles in longevity, aging‐associated diseases, and response to stresses (Bonkowski & Sinclair, 2016; Donmez & Guarente, 2010; Haigis & Sinclair, 2010; Imai & Guarente, 2014; Wątroba et al., 2017). Increased sirtuin activity is associated with the delayed onset of age‐related diseases, including cancer, cardiovascular disease, and diabetes (Banks et al., 2008; Hubbard & Sinclair, 2014; Kanfi et al., 2010; Kugel et al., 2016; Pfluger et al., 2008), and prolonged longevity in some circumstances (Kanfi et al., 2012; Mercken et al., 2014; Mitchell et al., 2014; Satoh et al., 2013). These beneficial effects might be attributable to the action of sirtuins in mitochondrial biogenesis, increased resistance against oxidative stress, and anti‐inflammatory effects (Donmez & Guarente, 2010; Finkel et al., 2009; Haigis & Sinclair, 2010; Hirschey et al., 2010; Kincaid & Bossy‐Wetzel, 2013; Rardin et al., 2013). It has been postulated that manipulation of sirtuin expression and/or activity could represent a new therapeutic approach for the prevention and treatment of osteoporosis. In this review paper, we present a comprehensive updated review of the current knowledge on sirtuin biology, focusing specifically on their roles in bone homeostasis and osteoporosis, and the potential pharmacological interventions that could target sirtuins for the treatment of osteoporosis.

## 2 MOLECULAR MECHANISM OF OSTEOPOROSIS

Osteoporosis is the most common bone remodeling disease, and the resulting increased risk of fragility fractures is of great concern (Pouresmaeili et al., 2018). Notably, the risk of fracture doubles with every 10% of bone mass lost (Rodan & Martin, 2000). The number of people who experience hip fracture in the United States has been estimated to reach over 6.26 million by 2050, resulting in dramatically high morbidity and healthcare costs (Gullberg et al., 1997). In China, there are currently over 60 million individuals diagnosed with osteoporosis, and the prevalence is estimated to be 6.49% and 29.13% for men and women aged 50 years or older, respectively (Zeng et al., 2019). Considering the huge social and economic burden associated with osteoporosis, there is an urgent need for a better understanding of the molecular mechanism underlying osteoporosis which might provide a scientific basis for the development of more effective therapies.

### 2.1 Mitochondrial dysfunction and oxidative stress

Recent clinical and preclinical animal studies have demonstrated an association between increased mitochondrial damage and osteoporotic bone loss (Trifunovic & Larsson, 2008; Trifunovic et al., 2004; Varanasi et al., 1999). Mice lacking HTRA2/OMI, an mitochondrial adenosine triphosphate (ATP)‐independent serine protease, exhibited elevated mtDNA deletions and severe osteoporosis (Kang et al., 2013). Impaired ATP production, caused by a dysfunctional mitochondrial transcription factor A (Tfam) gene, led to increased bone resorption (Miyazaki et al., 2012). Mitophagy and the unfolded protein response (UPR^mt^) are important for mitochondrial homeostasis (Fang et al., 2014; Mouchiroud et al., 2013b), whereas their disruption leads to severe bone loss, which is closely associated with impaired mitochondrial function (Wang et al., 2020; Zainabadi, Liu, Caldwell, et al., 2017). Furthermore, in vitro cellular and in vivo animal studies have demonstrated a link between mitochondria‐derived reactive oxygen species (ROS) and osteoporosis (Manolagas, 2010; Treiber et al., 2011; Yang et al., 2014). The skeletal changes in aging mice were accompanied by a progressive increase in ROS levels in the bone tissues (Almeida et al., 2007), which could adversely influence the survival and differentiation of osteoblasts and osteocytes from their corresponding progenitor cells. Mice with homozygous superoxide dismutase 2 (SOD2) deficiency in connective tissue (established using Col1α2‐Cre) exhibited decreased bone mineral density (BMD) associated with increased ROS levels (Treiber et al., 2011). Specific deletion of SOD2 in osteocytes caused remarkable bone loss in an age‐dependent manner, which was associated with the decreased number of osteocytes and disorganization of osteocyte canalicular networks resulting from increased ROS levels (Kobayashi et al., 2015). High levels of hydrogen peroxide (H_2_O_2_) in osteoblastic cells resulted in apoptosis and initiated osteoporosis by impairing osteoblast formation (Treiber et al., 2011; Yang et al., 2014). Conversely, accumulation of ROS induced osteoclast proliferation and facilitated osteoclast differentiation (Baek et al., 2010; Bartell et al., 2014; Garrett et al., 1990; Treiber et al., 2011; Yang et al., 2014). Loss of forkhead box O (FoxO) function in osteoclasts in mice increased osteoclast numbers and resulted in bone loss due to accumulation of intracellular H_2_O_2_ (Bartell et al., 2014). Attenuation of H_2_O_2_ generation in cells of the osteoclast lineage (using the lysozyme M promoter) abolished the loss of cortical bone following ovariectomy (OVX) by decreasing osteoclast numbers (Ucer et al., 2017). Collectively, factors modulating mitochondrial function and antioxidant defense could be potential targets for interventional treatment or prevention of osteoporosis.

### 2.2 Cellular senescence

Various intra‐ and extracellular stresses, such as DNA damage, oncogenic insults, reactive metabolites, and proteotoxic stress, lead to cellular senescence (LeBrasseur et al., 2015; Swanson et al., 2013; Tchkonia et al., 2013; Zhu et al., 2014), in which cells stop dividing, undergo distinct genetic and phenotypic changes, and develop a senescence‐associated secretory phenotype (SASP; Blasco et al., 2013; Campisi, 2013). SASP is characterized by the secretion of pro‐inflammatory cytokines, chemokines, and extracellular matrix‐degrading proteins, which have deleterious paracrine and systemic effects (Acosta et al., 2013; Coppé et al., 2010; Nelson et al., 2012; Xu, Palmer, et al., 2015). By secreting damaging factors to neighboring cells, the SASP induces cellular senescence in normal cells adjacent to the senescent cells via the bystander effect (Nelson et al., 2012). Reducing the number of senescent cells by either genetic (*INK*‐*ATTAC* transgenic mice) or pharmacological (long‐term senolytic treatment) clearance of *p16* ^Ink4a^‐expressing senescent cells has been shown to extend the life span and prevent the development of multiple aging‐related comorbidities in both prematurely and naturally aged mice (Baker et al., 2011; Roos et al., 2016; Xu, Palmer, et al., 2015). A recent study comparing young (6‐month‐old) and aged (24‐month‐old) mice demonstrated that osteocytes and myeloid cells were the two main senescent cells in the bone microenvironment (Farr et al., 2016). Increased osteocyte senescence was further confirmed by another study that showed higher expression of the senescence markers γH2AX and p16^Ink4a^, and several SASP markers in osteocytes in 21‐month‐old in comparison with their expression levels in 7‐month‐old mice (Piemontese et al., 2017). A subsequent study from the same group demonstrated similar findings in osteoprogenitors. Specifically, a decreasing number of osterix (Osx)‐expressing osteoprogenitor cells were observed with increasing age in mice and were associated with increased expression of senescence markers (Kim, Chang, et al., 2017). Furthermore, bone marrow stromal cells from old mice also exhibited elevated expression of SASP genes (Kim, Chang, et al., 2017; Sui et al., 2016). In addition to these findings on cellular senescence in age‐related bone loss, increased cellular senescence in bone cells was also associated with glucocorticoid‐induced (Leclerc et al., 2004; Li et al., 2012) and unloading‐induced (Okazaki et al., 2004; Sakai et al., 2002) bone loss. The causal relationship between cellular senescence and age‐related bone loss was evidenced by the higher bone mass and strength observed in aged mice after the elimination of senescent cells in bone tissue by activating transduced suicide genes or administrating senolytics or a JAK inhibitor (Farr et al., 2017; Kim, Chang, et al., 2017). It has been proposed that SASP‐associated factor secretion from the senescent osteoblasts and osteocytes could lead to increased bone resorption and reduced bone formation. Thus, targeting senescent osteocytes represents a novel treatment for age‐related bone loss. Furthermore, cell cycle arrest is part of cellular senescence, which limits osteoblast differentiation of skeletal progenitor cells and contributes to age‐related bone loss (Wang et al., 2012). Future in‐depth studies are needed to investigate the mechanisms by which cellular senescence could impact bone remodeling. In view of current evidence demonstrating beneficial effects, eliminating senescent cells in the bone microenvironment represents a novel strategy that may potentially be used for the prevention and treatment of osteoporosis.

### 2.3 Inflammation

Under the influence of lifelong exposure to chronic antigenic load and oxidative stress during aging, a variety of cytokines, including interleukin 6 (IL‐6), tumor necrosis factor alpha (TNF‐α), and interleukin 1 (IL‐1), are elevated and play direct roles in the pathogenesis of age‐related diseases, including atherosclerosis, Alzheimer's disease and diabetes (Bruunsgaard, 2002; Ferrucci & Fabbri, 2018; Sarkar & Fisher, 2006; Yuan et al., 2019). Recent emerging evidence suggests that osteoporosis and other age‐related disorders could, to certain extent, be considered as inflammatory diseases (Arron & Choi, 2000; Lorenzo, 2000). Clinical studies have reported an association between an increased risk of developing osteoporosis and inflammatory conditions, such as rheumatoid arthritis, ankylosing spondylitis, and inflammatory bowel disease (Bultink et al., 2005; Haugeberg et al., 2004; Mikuls et al., 2005; Mitra et al., 2000; Moschen et al., 2005). Inflammatory cytokines, such as TNF‐α, IL‐1, and IL‐6, are elevated and play critical roles in these conditions (Ishihara & Hirano, 2002; Manolagas & Jilka, 1995; Moschen et al., 2005). Specifically, the aforementioned cytokines promote osteoclast differentiation and activation, which has been linked to accelerated bone loss in various bone disorders including postmenopausal osteoporosis, Paget's disease, and idiopathic osteoporosis (Kim et al., 2009; Manolagas & Jilka, 1995; Moffett et al., 2005; Pacifici et al., 1989; Wei et al., 2005; Yun & Lee, 2004). In women, the most common cause of postmenopausal osteoporosis is estrogen depletion, which results in elevated levels of pro‐inflammatory and pro‐osteoclastic cytokines (Ershler et al., 1997; Liu et al., 2005; Pfeilschifter et al., 2002; Scheidt‐Nave et al., 2001). These findings support the observation that inflammation could exert significant influence on bone turnover and contribute to the development of osteoporosis.

## 3 BIOLOGICAL FUNCTIONS OF SIRTUINS

The first member of the sirtuin family, yeast Sir2 (silent information regulator 2), was isolated in a screening for silencing factors in budding yeast thirty years ago (Rine et al., 1979). Subsequent studies revealed that SIR2‐like genes, also known as sirtuins, play a key role in prolonging the life span of lower organisms (Kaeberlein et al., 1999; Kennedy et al., 1995). The important discovery that life span extension by caloric restriction is abolished in sirtuin‐deficient yeast has inspired multiple studies on mammalian sirtuins (Guarente & Picard, 2005; Boily et al., 2008). To date, seven mammalian homologs (SIRT1‐SIRT7) of nicotinamide adenine dinucleotide (NAD^+^)‐dependent lysine deacetylases have been identified and found to be important in the promotion of DNA repair, antioxidant defense, mitochondrial biogenesis, and anti‐inflammatory effects (Donmez & Guarente, 2010; Finkel et al., 2009; Haigis & Sinclair, 2010). These sirtuins differ from each other in tissue distribution, subcellular localization, enzymatic activity, and target proteins (Table 1). SIRT1 is predominantly located in the nucleus and cytosol and modulates various transcription factors, such as tumor protein p53 (p53; Vaziri et al., 2001), nuclear factor κ‐light‐chain‐enhancer of activated B cells (NF‐κB; Yeung et al., 2004), FoxO (Mouchiroud et al., 2013a; Shan et al., 2017) and peroxisome proliferator‐activated receptor γ coactivator 1α (PGC1α; Rodgers et al., 2005). SIRT2 is mostly found in the cytoplasm, whereas SIRT3, SIRT4, and SIRT5 are located in the mitochondria and contribute to the regulation of ATP production, antioxidant defenses, energy metabolism, and cell signaling (Haigis et al., 2006; Hirschey et al., 2010; Kincaid & Bossy‐Wetzel, 2013; Rardin et al., 2013). SIRT6 and SIRT7 are primarily localized in the nucleus and regulate the response to and repair of damaged DNA (Li et al., 2016; McCord et al., 2009).

**TABLE 1:** Location, substrates, functions, and enzyme activities of different sirtuins

Sirtuin	Subcellular localization	Main substrates	Functions	Enzyme activity
SIRT1	Nucleus and cytoplasm	p53, histones H1, H3, and H4, FOXO1/3/4, p300, NF‐κB, PGC1α, LKB1, PPARγ, ATG5/7/8, c‐Jun, c‐Myc, MyoD, MEF2, Ku‐70, XPA, XPC, NBS1, HSF1, BCL6, SMAD3/7, MLH1, PMS2, HIF, PTEN, AR, β‐catenin, DNMT1, AceCS1, eNOS, ING3, cyclin A2, UPC2, ACS1, CRTC2, PGAM‐1, SREBP, LXR, and FXR	Cell survival, life span regulation, metabolism regulation, and inflammation, oxidative stress response and mitochondrial biogenesis	Deacetylase
SIRT2	Nucleus and cytoplasm	α‐tubulin, Histone H3 and H4, HDAC6, p65, HOXA10, CDK1, FOXO1, FOXO3, and p300	Cell cycle regulation, neurodegeneration, and tumor suppression/promotion	Deacetylase
SIRT3	Mitochondria, nucleus and cytoplasm	LCAD, ACS2, IDH2, Ku70, FOXO3a, MnSOD, MRPL10, LCAD, HMG‐CoA2, SDH, NADH, LKB1, and OTC	Regulation of mitochondrial metabolism, protection against oxidative stress, tumor suppression	Deacetylase
SIRT4	Mitochondria	GDH, MCD, PDH, ANT2, ANT3, and IDE	Regulation of mitochondrial metabolism, amino acid catabolism, and tumor suppression	Deacetylase ADP‐ribosylase Lipoamidase
SIRT5	Mitochondria	Cyt c, CPS1, and UOX	Apoptosis, urea cycle, fatty acid metabolism, and amino acid metabolism	Deacetylase Desuccinylase Demalonylase
SIRT6	Nucleus	Histone H3, TNF‐α, PAPR1, HIF1α, NPM1, KAP1, and GCN5	Genome stability, DNA repair, glucose and lipid metabolism, and inflammation	Deacetylase ADP‐ribosylase
SIRT7	Nucleus	FOXO3, NPM1, PGK1, CDK9, DDB1, SMAD4, RNA polymerase I, and p53	Regulation of rRNA transcription, cell cycle regulation, and ribosome biogenesis	Deacetylase

p53, tumor protein p53 ; FOXO1/3/4, forkhead box O‐1/3/4; p300, histone acetyltransferase p300; NF‐κB, nuclear factor kappa‐light‐chain‐enhancer of activated B cells; PGC1α, peroxisome proliferator‐activated receptor alpha; LKB1, liver kinase B1; PPARγ, peroxisome proliferator‐activated receptor gamma; ATG5/7/8, autophagy‐related gene 5/7/8; MyoD, myogenic differentiation 1; MEF2, myocyte enhancer factor 2; XPA, xeroderma pigmentosum, complementation group A; XPC, xeroderma pigmentosum, complementation group C; NBS1, Nijmegen breakage syndrome 1; HSF1, heat shock transcription factor 1; BCL6, B‐cell lymphoma 6 protein; SMAD3/7, SMAD family member 3/7; MLH1, MutL homolog 1; PMS2, PMS1 homolog 2; HIF, hypoxia‐inducible factor; PTEN, phosphatase and tensin homolog; AR, androgen receptor; DNMT1, DNA methyltransferase 1; AceCS1, Acyl‐CoA synthetase short‐chain family member 1; eNOS, endothelial nitric oxide synthase; ING3, inhibitor of growth family member 3; UPC2, ultraperformance convergence chromatography; ACS1, acetyl‐CoA synthetase 1; CRTC2, CREB‐regulated transcription coactivator 2; PGAM‐1, phosphoglycerate mutase‐1; SREBP, sterol regulatory element‐binding protein; LXR, liver X receptor; FXR, farnesoid X receptor; HDAC6, histone deacetylase 6; HOXA10, homeobox protein A10; CDK1, cyclin‐dependent kinase 1; LCAD, long‐chain‐specific acyl coenzyme A dehydrogenase; ACS2, acetyl‐CoA synthetase 2; IDH2, isocitrate dehydrogenase 2; Ku70, lupus Ku autoantigen protein p70; MnSOD, mitochondrial manganese superoxide dismutase; MRPL10, mitochondrial ribosomal protein L10; HMG‐CoA2, 3‐hydroxy‐3‐methylglutaryl CoA synthase 2; SDH, succinate dehydrogenase; NADH, nicotinamide adenine dinucleotide; OTC, ornithine transcarbamoylase; GDH, glutamate dehydrogenase; MCD, malonyl CoA decarboxylase; PDH, pyruvate dehydrogenase; ANT2, adenine translocator 2; ANT3, adenine translocator 3; IDE, insulin‐degrading enzyme; Cyt c, cytochrome c; CPS1, carbamoyl phosphate synthetase 1; UOX, urate oxidase; TNF‐α, tumor necrosis factor α; PAPR1, poly (adenosine diphosphate‐ribose) polymerase‐1; HIF1α, hypoxia‐induced factor 1; NPM1, nucleophosmin 1; KAP1, the Krüppel associated box (KRAB)‐associated protein‐1; GCN5, general control non‐repressed protein 5 (an acetyltransferase); PGK1, phosphoglycerate kinase 1; CDK9, cyclin‐dependent kinase 9; DDB1, damage specific DNA binding protein 1; SMAD4, mothers against decapentaplegic homolog 4.

A detailed discussion of the biological function of each sirtuin in mitochondrial function, antioxidative stress, inflammation, and cellular senescence has been covered extensively in the literature (Donmez & Guarente, 2010; Haigis & Sinclair, 2010; Kitada et al., 2019; Satoh et al., 2011), suggesting a link between sirtuins and osteoporosis. This review focuses more on the biological functions of sirtuins in bone homeostasis.

## 4 SIRTUINS IN BONE BIOLOGY

### 4.1 SIRT1

Among the seven sirtuins, SIRT1 is the most studied and plays critical role in normal skeletal development and homeostasis (Zainabadi, 2019). This review attempts to provide more updated information regarding the role of SIRT1 in bone biology and osteoporosis development.

**FIGURE 1:**
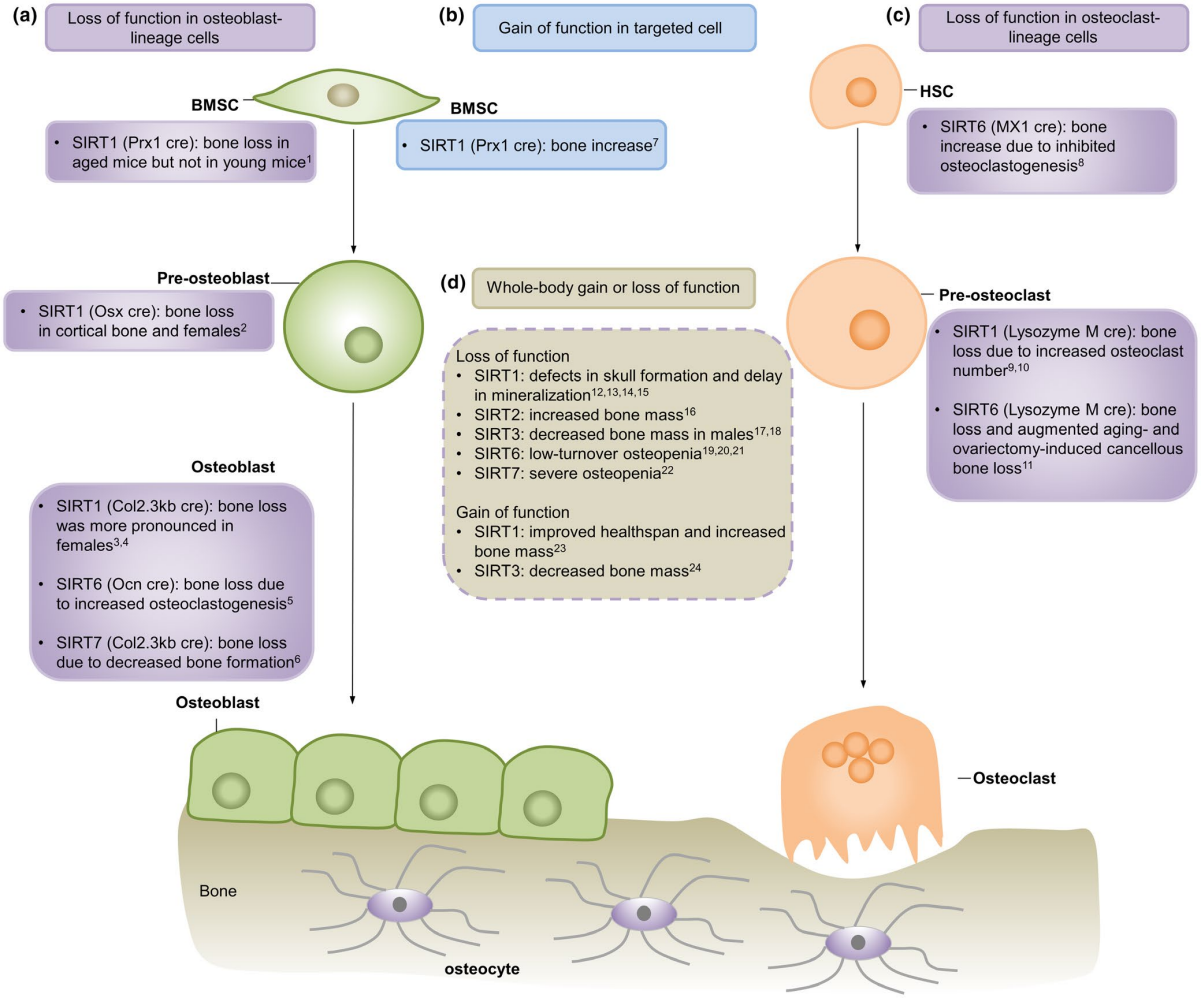
Effects of sirtuins on bone mass as determined from studies using knockout and transgenic mouse models. (a) Effect of loss of function of SIRT1‐SIRT7 in osteoblast lineage cells on bone mass. Target cells included BMSCs, pre‐osteoblasts, and osteoblasts. (b) Effect of gain of function of SIRT1 in BMSCs on bone mass. (c) Effect of loss of function of SIRT1‐SIRT7 in osteoclast‐lineage cells on bone mass. Target cells included HSC and preosteoclasts. (d) Effect of whole‐body gain or loss of function of SIRT1‐SIRT7 on bone mass as determined using mouse models with global SIRT1‐SIRT7 overexpression or knockout. BMSCs, bone mesenchymal stromal cells; Osx, osterix; Ocn, osteocalcin; HSC, hematopoietic stem cell. ^1^(Simic et al., 2013), ^2^(Iyer et al., 2014), ^3^(Zainabadi, Liu, Caldwell, et al., 2017), ^4^(Edwards et al., 2013), ^5^(Kim et al., 2020), ^6^(Fukuda et al., 2018), ^7^(Sun et al., 2018), ^8^(Park et al., 2016), ^9^(Zainabadi, Liu, Caldwell, et al., 2017), ^10^(Edwards et al., 2013), ^11^(Moon et al., 2019), ^12^(Cheng et al., 2003), ^13^(Lemieux et al., 2005), ^14^(McBurney et al., 2003), ^15^(Zainabadi, Liu, Guarente, 2017), ^16^(Jing et al., 2019), ^17^(Gao et al., 2018), ^18^(Huh et al., 2016), ^19^(Sugatani et al., 2015), ^20^(Zhang, Ryu, et al., 2016), ^21^(Zhang et al., 2018), ^22^(Fukuda et al., 2018), ^23^(Herranz et al., 2010), and ^24^(Ho et al., 2017)

Pioneering work on the role of SIRT1 in bone biology was conducted in SIRT1 global knockout (KO) mice (Cheng et al., 2003; Lemieux et al., 2005; McBurney et al., 2003). Compared with wild‐type control mice, SIRT1 KO embryos and newborn pups were smaller at birth and exhibited higher rates of perinatal lethality and notable developmental defects of the retina and heart (Figure 1). These mice also had craniofacial abnormalities, including defects in the development and closure of craniofacial sutures, abnormal palate architecture, and instances of exencephaly (Cheng et al., 2003; McBurney et al., 2003). Furthermore, SIRT1 KO pups exhibited delayed mineralization of the skull, vertebrae, and digits (Lemieux et al., 2005). Likewise, deletion of SIRT1 in adult mice showed a significant reduction in both trabecular and cortical bone irrespective of sex (Mercken et al., 2014; Zainabadi, Liu, Caldwell, et al., 2017). These results suggest an important role of SIRT1 in bone development and remodeling. However, it is difficult to interpret these findings separately from the frequently associated gross developmental abnormalities, including smaller size, sterility, and high rates of postnatal lethality (Cheng et al., 2003; McBurney et al., 2003). Additional observations of adult heterozygous KOs showed a significant reduction in both trabecular and cortical bone mass in long bones (Cohen‐Kfir et al., 2011) without developmental abnormalities, thus supporting the hypothesis that SIRT1 plays a role in bone remodeling. Interestingly, the phenotypic changes appear to be sex‐ and age‐specific, as indicated by the more pronounced alterations in female and young mice (1‐month‐old). Furthermore, it is evidenced that SIRT1 could influence bone remodeling via hormone and endocrine signaling pathways and the somatotropic axis (Cohen et al., 2009; Kolthur‐Seetharam et al., 2009; Toorie et al., 2016). The possible link between SIRT1 and steroid hormone signaling pathways is verified by the upregulation of SIRT1 expression upon estrogen treatment. In contrast, OVX caused a decline in SIRT1 expression (Elbaz et al., 2009; Shakibaei et al., 2012; Artsi et al., 2014; Wang et al., 2017).

To examine whether SIRT1 exerts its effect on bone metabolism through direct actions on bone cells or via intermediate hormone signaling, a number of mouse models with specific deletion of SIRT1 in different bone cells, including osteoblasts, osteoclasts, and mesenchymal stem cells, have been developed (Edwards et al., 2013; Simic et al., 2013; Zainabadi, Liu, Caldwell, et al., 2017; Figure 1). Of note, deletion of SIRT1 in osteoblasts (ObcKOs) using the collagen type 1 2.3 kb promoter and in osteoclasts (OccKOs) using the lysozyme M promoter showed lower trabecular bone mass (Zainabadi, Liu, Caldwell, et al., 2017). However, double deletion of SIRT1 in both osteoblasts and osteoclasts did not result in a more severe bone loss phenotype (Zainabadi, Liu, Caldwell, et al., 2017). Mechanistic studies revealed that the bone loss phenotype of ObcKOs was associated with a lower osteoblast number and reduced bone formation rate (BFR), while increased osteoclast number was observed in OccKOs. These findings support the hypothesis that SIRT1 can exert a direct effect on osteoblast and osteoclast.

Furthermore, deletion of SIRT1 in osteoprogenitors using the Osx promoter resulted in lower cortical bone thickness at the endocortical surface as a result of decreased bone formation, but had no effect on trabecular bone mass (Iyer et al., 2014). Similar phenotypic changes were observed in mice with the deletion of SIRT1 in earlier osteoblast precursors using the Prx1 promoter (Simic et al., 2013). These results, along with the finding that cortical bone was unaffected in mice lacking SIRT1 in mature osteoblasts (Edwards et al., 2013), suggest that SIRT1 could protect the cortical and trabecular bone compartments via actions on different osteoblast populations (i.e., early versus late in the osteoblast lineage) through different mechanisms. In contrast to the bone loss phenotype in female mice, bone loss was not observed in male mice. A potential explanation could be the existence of a cross‐talk between SIRT1 and estrogen receptor α (ERα; Elangovan et al., 2011; Ji Yu et al., 2011). The bone loss phenotype observed using Prx1‐Cre was more pronounced in aged mice (26‐month‐old) than in young mice (2‐month‐old; Simic et al., 2013), which was accompanied by a marked decrease in the number of marrow‐derived mesenchymal stem cells (MSCs). These results suggest that in addition to its role in regulating the osteogenic differentiation potential to osteoblast, SIRT1 also positively regulates MSC self‐renewal (Yoon et al., 2014). On the other hand, chondrocyte‐specific deletion of SIRT1 using the collagen II promoter resulted in growth retardation and shorter bones, which was associated with decreased chondrocyte proliferation and hypertrophy (Jin et al., 2019). In addition, SIRT1 has been shown to promote cartilage‐specific gene expression (Dvir‐Ginzberg et al., 2008) and protect chondrocyte against radiation‐induced senescence (Hong et al., 2010). These findings were consistent with reduced levels of SIRT1 measured in human osteoarthritis (OA) cartilage (Fujita et al., 2011; Takayama et al., 2009), suggesting a potential protective role of SIRT1 in chondrocytes, which was further evidenced by the accelerated development of OA in mice lacking SIRT1 in chondrocyte (Matsuzaki et al., 2014). However, a recent finding demonstrated an increased ratio of proliferative‐to‐hypertrophic zone following SIRT1 deletion in chondrocyte, suggesting SIRT1 inhibits chondrocyte proliferation (Shtaif et al., 2020). Possible reason for the discrepancy could be due to the differences in the genetic models, which requires further investigation. In addition to its role in chondrogenesis, the absence of SIRT1 in chondrocytes also resulted in decreased trabecular and cortical bone mineralization, which was attributed to the disorganized epiphyseal growth plate in chondrocyte‐specific KO mice (Shtaif et al., 2020).

In addition to the KO models, SIRT1 overexpression mouse models have also been developed to study the impact of SIRT1 activation on bone homeostasis (Herranz et al., 2010; Sun et al., 2018; Wang et al., 2019). Encouragingly, these transgenic mice showed 40%–50% higher bone mass than that of control mice at 2.5 years of age (Herranz et al., 2010), as well as improved healthy aging. More recently, overexpression of SIRT1 in cells of the mesenchymal lineage (using the Prx1 promoter) showed increased bone volume in both young mice (1‐month‐old) and aged mice (18‐month‐old), which was associated with increased osteoblast and decreased osteoclast numbers (Sun et al., 2018; Wang et al., 2019). These effects were achieved by restoring the redox balance in MSCs. These preclinical findings suggest that SIRT1 could be a potential target for the development of novel anti‐osteoporotic therapy.

### 4.2 SIRT2

SIRT2 KO mice developed normally at a young age but exhibited tumorigenesis in multiple tissues at an advanced age, suggesting an essential role of SIRT2 in maintaining genetic stability and repressing tumor formation (Kim et al., 2011). Among all reported SIRT2 KO studies (Beirowski et al., 2011; Jing et al., 2019; Kim et al., 2011), one related to skeleton changes was identified (Jing et al., 2019) and is included in the present review. Compared with the wild‐type control group, rats with SIRT2 deletion (SIRT2 KO) exhibited a higher total bone volume fraction, trabecular bone mineral density, and trabecular number at the age of 36 weeks, which was not apparent at the age of 12 weeks. In an in vitro study, it was demonstrated that inhibiting SIRT2 expression using AGK2 could suppress osteoclastogenic differentiation (Jing et al., 2019). Further investigation is required to delineate how SIRT2 regulates osteoclast differentiation and activity, and its effect on age‐related bone loss.

### 4.3 SIRT3

SIRT3, a mitochondrial NAD^+^‐dependent protein deacetylase (Ansari et al., 2017; Lombard et al., 2007), was found to be associated with long life span in humans and has, thus, attracted public and research interest (Bellizzi et al., 2005; Rose et al., 2003). An initial study showed that SIRT3 KO mice appeared to be structurally normal, without significant changes in body composition and BMD at a young age (Lombard et al., 2007). However, aging‐related health problems such as metabolic syndrome, cancer, and cardiovascular and neurodegenerative diseases were observed earlier in the SIRT3 KO mice (McDonnell et al., 2015; Van de Ven et al., 2017). SIRT3 KO mice exhibited lower trabecular bone mass in long bones at a young age (8‐week‐old), indicating a positive role of SIRT3 during the development of peak bone mass (Gao et al., 2018; Huh et al., 2016). However, such differences became more subtle in SIRT3 KO mice reaching adulthood (6‐month‐old; Ho et al., 2017). In contrast, mice with global SIRT3 overexpression had normal bone mass at young age (3‐month‐old) but exhibited lower bone mass at an older age (13‐month‐old; Ho et al., 2017). Evidence from mice with different SIRT3 expression levels suggests the likelihood of an age‐dependent effect of SIRT3 on bone, but further investigations with cell/tissue‐specific SIRT3 KOs and wider age spectrums are warranted to unravel the biological function of SIRT3 on bone metabolism and aging.

### 4.4 SIRT6

SIRT6 is involved in the regulation of chromatin and is shown to play a number of roles in metabolism, aging, and diseases (Kanfi et al., 2008; Kugel & Mostoslavsky, 2014; Mostoslavsky et al., 2006; Mu et al., 2018). The first study of bone in SIRT6 KO mice demonstrated a progeroid degenerative syndrome including reduced size, lordokyphosis, and severe osteopenia with a 30% reduction in BMD (measured with dual‐energy X‐ray absorptiometry [DXA]; Mostoslavsky et al., 2006). A more recent investigation of young SIRT6 KO mice showed significant deficiencies in both trabecular BMD and cortical bone volume compared with those of control mice (Sugatani et al., 2015; Zhang, Cui, et al., 2016; Zhang et al., 2018), which was associated with impaired bone formation. The effects of SIRT6 on bone resorption remain controversial, with only one report of impaired osteoclast function (Sugatani et al., 2015). In contrast, most other studies have reported increased osteoclastogenesis reported (Zhang et al., 2016a, 2018). Interpretation of these findings is difficult due to the extremely small size of the tested animals and the overall poor health condition of the SIRT6 KO mice.

To better understand the exact molecular role of SIRT6 in bone homeostasis, several osteoblast lineage‐ and osteoclast lineage‐specific SIRT6 KO mice have been developed. Specifically, osteoblast lineage‐specific SIRT6 KO mice established using the osteocalcin promoter exhibited osteopenia, which was attributed to a paracrine activation of osteoclastogenesis due to decreased osteoprotegerin (OPG) levels without changes in osteoblast function (Kim et al., 2020). Targeted deletion of SIRT6 in hematopoietic cells (using the Mx1 promoter), including osteoclast precursors, resulted in increased bone volume, which could be due to the decreased number of osteoclasts (Park et al., 2016). In contrast, myeloid SIRT6 deletion (using the lysozyme M promoter) resulted in decreased trabecular bone mass in aged mice (20‐month‐old). This age‐related change in phenotype was associated with a twofold increase in the number of mature osteoclasts in SIRT6 KO mice (Moon et al., 2019). These results, along with the controversial findings on the effects of SIRT6 on osteoclasts, suggest that a more in‐depth elucidation of the role of SIRT6 in skeletal homeostasis is required, such as in cKO and transgenic animal models.

### 4.5 SIRT7

SIRT7 acts as a histone deacetylase with an important role in the DNA damage response and cell survival (Li et al., 2016), as evidenced by the genomic instability and premature aging phenotypes of SIRT7‐deficient mice (Paredes & Chua, 2016). It has been proposed that upon DNA damage, SIRT7 is mobilized onto chromatin and deacetylates ataxia–telangiectasia mutated (ATM) to control proper DNA damage repair. Deletion of SIRT7 induces impaired DNA damage repair due to persistent ATM activation (Tang et al., 2019). Furthermore, SIRT7 promotes DNA repair by direct H3K18Ac deacetylation at DNA double‐strand break (DSB) sites and then triggers recruitment of non‐homologous end joining (NHEJ) repair factors, which are speculated to be the primary pathway for the repair of DSBs (Chang et al., 2017; Vazquez et al., 2016). SIRT7 KO mice were found to have deficiencies in embryonic and postnatal development, including elevated perinatal lethality and shorter life span with signs of progeroid‐like phenotypes including kyphosis, heart hypertrophy, hepatic steatosis, deafness, increased p16^INK4^ expression, and reduced circulating insulin‐like growth factor 1 (IGF‐1) protein (Ryu et al., 2014; Shin et al., 2013; Vakhrusheva et al., 2008; Vazquez et al., 2016). Analysis of adult female SIRT7 KO mice revealed significant deficiencies in both trabecular and cortical bone mass, which was associated with defects in osteoblast numbers and bone formation (Fukuda et al., 2018). Furthermore, tissue‐specific deletion of SIRT7 in osteoblasts (using the α1(I)‐collagen promoter) showed decreased trabecular bone mass and cortical bone thickness (Fukuda et al., 2018). Importantly, the phenotype of obcKOs was found to be associated with lower in vivo osteoblast numbers and reduced BFR, with no effect on osteoclastogenesis. The stimulatory effects of SIRT7 on osteoblasts might be due to its positive regulation of the transactivation activity of Osx through deacetylation of lysine 368 in its C‐terminal region. However, there is no available data on the influence of SIRT7 on osteoclast function, which requires further investigation.

## 5 POSSIBLE MECHANISMS UNDERLYING THE BIOLOGICAL ACTIONS OF SIRTUINS IN BONE

### 5.1 SIRT1

The above in vivo studies all point to the possible important roles of sirtuins in the regulation or modulation of different phases of bone remodeling. These findings are in line with the in vitro results.

**FIGURE 2:**
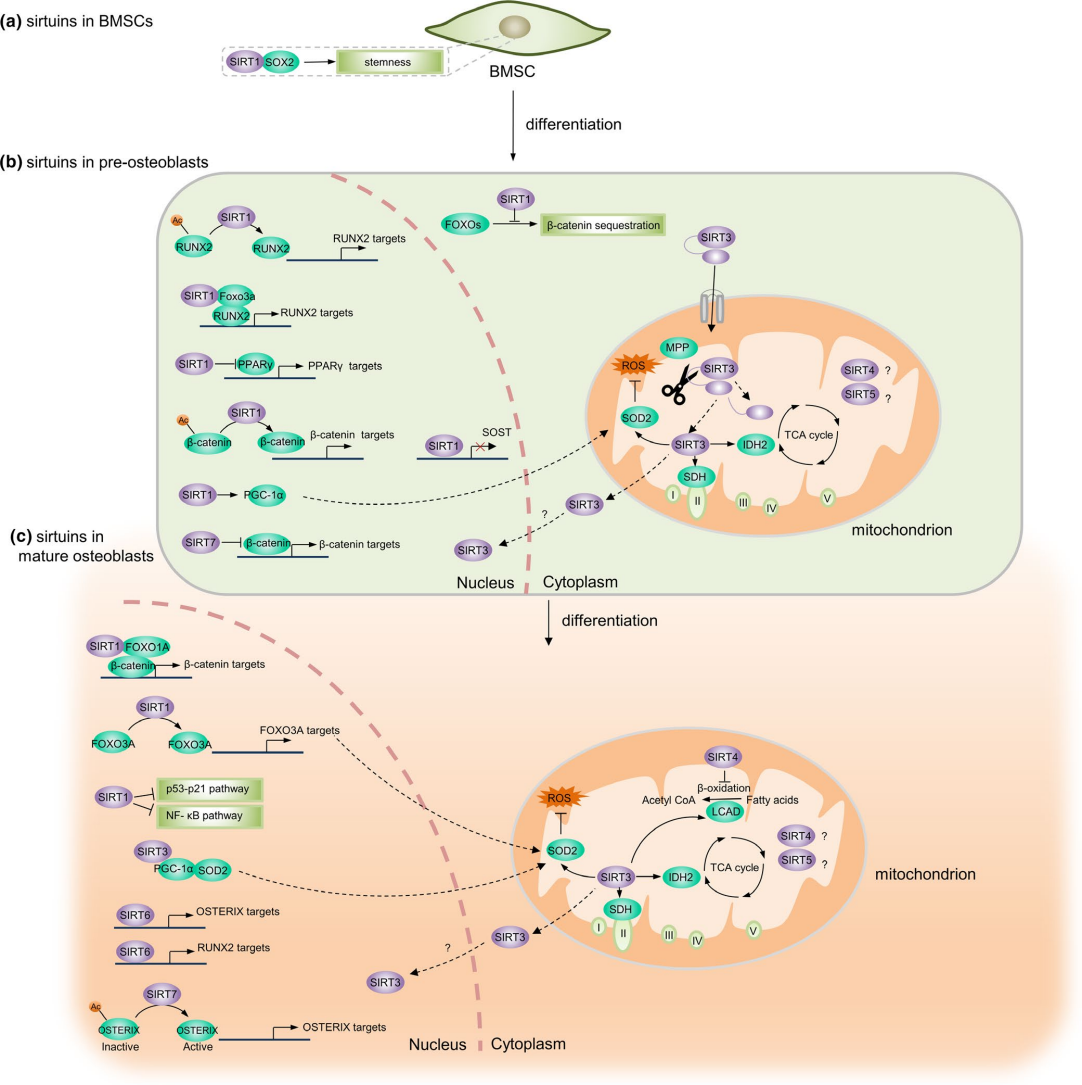
Sirtuins regulate BMSCs, osteogenic differentiation, and osteoblast function through multiple downstream targets. (a) SIRT1 maintains BMSC self‐renewal and stemness through its direct regulation of SOX2. (b) SIRT1 regulates osteogenic differentiation of BMSCs by directly or indirectly interacting with RUNX2, β‐catenin, SOD2, PPARγ, FOXOs, PGC1α, and SOST, thus modulating the expression of their target genes. SIRT3 decreases ROS production by stimulating SOD2, and SIRT3 also enhances cellular respiration by increasing the activities of complex I, complex II, complex III, and IDH2. (c) SIRT1 regulates the function of osteoblast by directly or indirectly regulating β‐catenin, FOXO3a, and the p53‐p21 and NF‐κB signaling pathways, modulating the expression of their targets. SIRT3 decreases ROS production by stimulating SOD2. SIRT6 interacts with RUNX2 and OSTERIX via deacetylating H3 K9 in their promoters and increasing the expression of their target genes. SIRT7 promotes the transcriptional activity of OSTERIX by deacetylating its promoters and increasing expression of its target genes. BMSCs, bone mesenchymal stromal cells; SOD2, sex‐determining region Y‐box 2; RUNX2, regulating runt‐related transcription factor 2; SOD2, superoxide dismutase 2; PPARγ, peroxisome proliferator‐activated receptor γ; FOXOs, forkhead box O transcription factors; PGC1α, peroxisome proliferator‐activated receptor γ coactivator 1α; SOST, sclerostin; ROS, reactive oxygen species; IDH2, isocitrate dehydrogenase 2; FOXO3a, forkhead transcription factor 3a; NF‐κB, nuclear factor kappa‐light‐chain‐enhancer of activated B cells; H3 K9, histone H3 at lysine 9; MPP, matrix processing peptidase; Ac, acetylation; SDH, succinate dehydrogenase; TCA, tricarboxylic acid cycle

**FIGURE 3:**
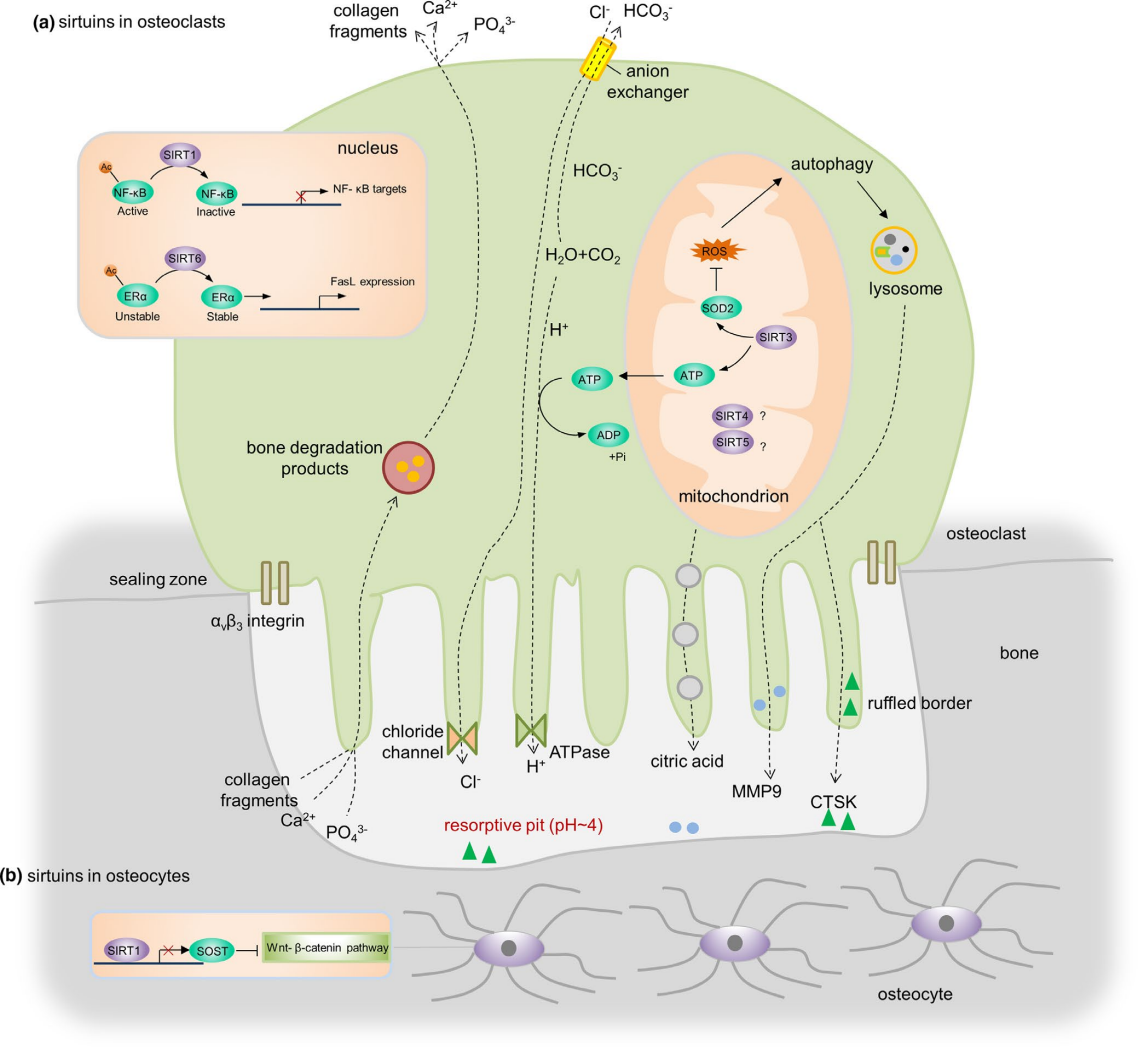
Sirtuins regulate osteoclast and osteocyte function through multiple downstream targets. (a) SIRT1 inhibits the activity of the NF‐κB protein complex through the deacetylation of lysine 310 on the p65 subunit and thus decreases the expression of its target genes. SIRT3 decreases ROS production by stimulating SOD2 in mitochondria. Excessive ROS levels stimulate autophagy and promote the release of proteolytic enzymes (e.g., CTSK, MMP9) from lysosomes to resorb bone. Furthermore, SIRT3 regulates ATP generation, which is required for the generation of hydrogen ions released through vacuolar ATPase proton pumps to create an acidic environment in the resorptive pit. SIRT6 deacetylates the ERα protein to prevent its proteasomal degradation and promotes transcription of FasL in preosteoclasts, resulting in osteoclast apoptosis. (b) SIRT1‐dependent deacetylation of the *Sost* promoter results in decreased sclerostin expression and enhanced WNT/β‐catenin signaling. NF‐κB, nuclear factor kappa‐light‐chain‐enhancer of activated B cells; ROS, reactive oxygen species; SOD2, superoxide dismutase 2; CTSK, cathepsin K; MMP9, matrix metalloproteinase‐9; ATP, adenosine triphosphate; ADP, adenosine diphosphate; ERα, estrogen receptor alpha; FasL, fas ligand

SIRT1 promotes self‐renewal and maintenance of MSCs through its direct regulation of the sex‐determining region Y‐box 2 (SOX2; Yoon et al., 2014; Figure 2a). SIRT1 has also been reported to promote osteoblast differentiation of MSCs by activating runt‐related transcription factor 2 (RUNX2) through direct deacetylation of RUNX2 or indirect modulation by forming a SIRT1‐forkhead box O3 (FOXO3A) complex (Shakibaei et al., 2012; Tseng et al., 2011; Zainabadi, Liu, & Guarente, 2017), and repressing peroxisome proliferator‐activated receptor γ (PPARγ; Bäckesjö et al., 2006; Picard et al., 2004; Figure 2b). In addition, SIRT1 has been shown to promote osteoblast activity by stimulating Wnt signaling through deacetylation effect directly (Simic et al., 2013) or indirect deacetylation of FoxOs, which prevents the association of FoxO with β‐catenin, thus resulting in increased expression of β‐catenin (Iyer et al., 2014). SIRT1 may also promote bone formation by decreasing the expression of SOST (Artsi et al., 2014; Cohen‐Kfir et al., 2011; Stegen et al., 2018), an inhibitor of bone formation, by modifying H3 K9 acetylation at the *Sost* promoter, silencing SOST expression. Additionally, SIRT1 activates PGC1α transcriptional activity to induce mitochondrial biogenesis and the induction of antioxidative enzymes, which can protect bone cells and inhibit the generation of mitochondrial ROS (Cantó et al., 2009; He et al., 2010; Yao et al., 2018). In addition to its stimulatory actions on osteogenic differentiation from bone mesenchymal stromal cells (BMSCs), SIRT1 has also been shown to play a role in mature osteoblasts (Figure 2c). Upregulation of SIRT1 inhibits H_2_O_2_‑induced osteoblast apoptosis via the FoxO1/β‑catenin pathway (Yao et al., 2018). Similarly, SIRT1 has been shown to suppress osteoblast apoptosis by inhibiting both the p53‐p21 and NF‐κB signaling pathways (Gu et al., 2019; Huang et al., 2012). Furthermore, SIRT1 has been shown to repress osteoclast differentiation through negative regulation of NF‐κB and positive regulation of FoxO transcription factors (Edwards et al., 2013; Kim et al., 2015; Figure 3a).

The roles of sirtuins in osteocytes are less studied to date. A recent study demonstrated that SIRT1‐dependent deacetylation of the *Sost* promoter resulted in decreased sclerostin expression and enhanced WNT/β‐catenin signaling, leading to increased bone mass in mice (Stegen et al., 2018; Figure 3b).

### 5.2 SIRT2‐7

To date, the roles of other sirtuins in bone cells in vitro have not been extensively studied. Inhibition of SIRT2 with its specific inhibitor, AGK2, suppressed the differentiation of BMMs into osteoclasts by reducing the expression of c‐Fos and nuclear factor of activated T cells, cytoplasmic 1 (NFATc1; Jing et al., 2019). SIRT3 was required for osteogenic differentiation through positive regulation of SOD2 and maintenance of mitochondrial function in osteoblasts (Ding et al., 2017; Gao et al., 2018; Figure 2b). Despite its clear protective role in osteogenesis, the effect of SIRT3 on osteoclastogenesis still remains controversial. Deletion of SIRT3 in BMMs enhanced osteoclast differentiation through modulation of ROS, which stimulated the differentiation and activation of osteoclasts (Kim et al., 2017). These findings are in contrast to a recent finding that demonstrated that SIRT3 positively regulated osteoclastogenesis by activating the mammalian target of rapamycin (mTOR) pathway (Ho et al., 2017). In addition to the different experimental conditions and materials used, such as BMMs of different origins. Impaired ATP production following SIRT3 deficiency could also explain the above discrepancy due to the high energy demand of osteoclastic bone resorption (Ishii et al., 2009). In addition, autophagy is involved in osteoclastic bone resorption, which is regulated by ROS levels (Scherz‐Shouval & Elazar, 2011; Figure 3a). Deletion of SIRT6 in BMSCs resulted in impaired osteogenesis (Sun et al., 2014; Zhang, Cui, et al., 2016), whereas overexpression of SIRT6 impaired osteoblast differentiation of human MSCs (Xiao et al., 2019). The exact mechanism by which SIRT6 affects osteogenesis requires further investigation. SIRT6 has been shown to promote osteoblast activity through direct interaction with RUNX2 and Osx via deacetylating histone H3 at lysine 9 (H3 K9) in their promoters (Sugatani et al., 2015). Furthermore, SIRT6 has also been shown to regulate osteoclastogenesis. The absence of SIRT6 impaired osteoclastogenesis associated with increased OPG expression (Park et al., 2016; Sugatani et al., 2015), whereas SIRT6 deficiency was reported to induce activation of osteoclasts by inhibiting transcription of Fas ligand (FasL) in preosteoclasts, resulting in an increased number of osteoclasts (Moon et al., 2019). Details of the regulation mechanisms need to be further clarified. A more recent study showed that SIRT7 knockdown accelerated osteogenesis of human BMSCs, at least in part via the Wnt/β‐catenin signaling pathway (Chen et al., 2017). In this regard, it is noteworthy that SIRT7 acts on multiple cell types to regulate bone metabolism.

## 6 POTENTIAL THERAPEUTIC AGENTS TARGETING SIRTUINS FOR THE TREATMENT OF OSTEOPOROSIS

These findings open up a new line of investigation into the metabolic control of sirtuins and modulation of their activity by small molecules. The activation of sirtuins either by genetic or by pharmacological means extends life span and promotes the health of a wide variety of organisms (Kaeberlein et al., 1999; Kanfi et al., 2012; Mercken et al., 2014; Mitchell et al., 2014; Satoh et al., 2013), thus demonstrating the feasibility of developing drugs that target sirtuins in the treatment of osteoporosis.

### 6.1 SIRT1

**FIGURE 4:**
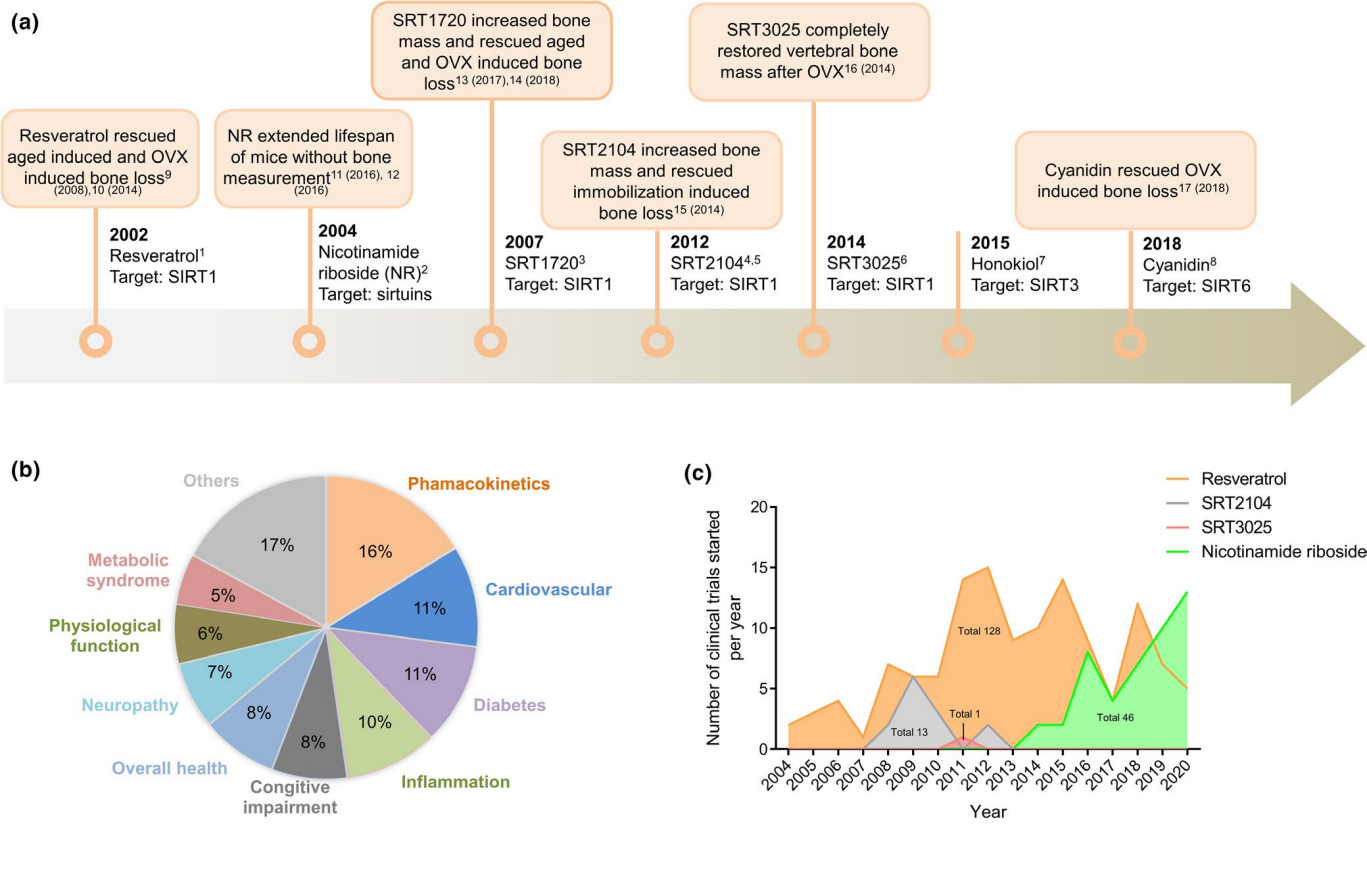
Timeline of the discovery of representative sirtuin‐activating compounds and their preclinical and clinical effects. (a) The effects of different sirtuin‐activating compounds on bone mass were determined using animal studies. (b) The chart shows the distribution and percentage of indications for clinical studies of sirtuin‐activating compounds registered on ClinicalTrials.gov, which were started between January 2004 and July 2020. We excluded studies with suspension, termination, and withdrawal statuses and those without FDA‐defined phases. Other diseases included Friedreich ataxia, pseudoachondroplasia, lymphangioleiomyomatosis, endometriosis, common cold, knee osteoarthritis, infertility, renal dysfunction, COVID‐19, cancer, allogeneic hematopoietic cell transplantation, and ataxia–telangiectasia. (c) The graph shows the number of studies of each sirtuin‐activating compound posted on ClinicalTrials.gov each year between 2004 and 2020, based on the start date. We excluded studies with suspension, termination, and withdrawal statuses. FDA, food and drug administration; COVID‐19, coronavirus disease 2019. ^1^(Howitz et al., 2003), ^2^(Bieganowski & Brenner, 2004), ^3^(Milne et al., 2007), ^4^(Hoffmann et al., 2013), ^5^(Libri et al., 2012), ^6^(Hubbard & Sinclair, 2014), ^7^(Pillai et al., 2015), ^8^(Rahnasto‐Rilla et al., 2018), ^9^(Pearson et al., 2008), ^10^(Feng et al., 2014), ^11^(H. Zhang, Ryu, et al., 2016), ^12^(Fang et al., 2016), ^13^(Zainabadi, Liu, Caldwell, et al., 2017), ^14^(Stegen et al., 2018), ^15^(Mercken et al., 2014), ^16^(Artsi et al., 2014), and ^17^(Cheng et al., 2018)

Resveratrol, the first generation of SIRT1 activator discovered in 2003, is a polyphenol found in nuts, grapes, and other plant sources, and can increase SIRT1 protein expression in a dose‐dependent manner (Kim et al., 2014) and extend the life span of yeast (Howitz et al., 2003). Cumulating evidence has demonstrated the protective effect of resveratrol on osteoporosis (Pearson et al., 2008). Mice treated with resveratrol for over 18 months showed moderate improvements in both trabecular and cortical bone mass, indicating protective effect of SIRT1 activation on aging‐related bone loss (Pearson et al., 2008; Figure 4a). Furthermore, resveratrol treatment protected against bone loss in OVX models of postmenopausal osteoporosis (Feng et al., 2014). In addition to the osteoporosis model, resveratrol administration for 10 weeks also increased bone mass and osteoblast number in young mice (12‐week‐old; Zhao et al., 2018). However, resveratrol may be able to activate SIRT3 (Chen et al., 2015) and SIRT5 (Gertz et al., 2012) as well as other non‐sirtuin targets, including AMPK (Fulco et al., 2008), F1‐ATPase (Gledhill et al., 2007), and PARp1 and Thr‐tRNA (Sajish & Schimmel, 2015). To avoid this, more specific and selective SIRT1 activators with a greater substrate‐binding affinity for SIRT1 have been developed.

SRT1720, SRT2104, and SRT3025 are representative sirtuin‐activating compounds (STACs) discovered in the past two decades. SRT1720 was found to be beneficial for preventing bone loss. For instance, femoral trabecular bone mass in aged mice (12‐month‐old) increased by 50% after five months’ treatment of SRT1720 (Zainabadi, Liu, Caldwell, et al., 2017). Similarly, administration of SRT1720 attenuated bone loss in OVX models (Zainabadi, Liu, Caldwell, et al., 2017). These benefits could be attributed to the stimulation of bone formation, inhibition of resorption, and reduction in sclerostin levels (Stegen et al., 2018). Among the various STACs, SRT2104 not only extended the life span in mice, but also enhanced BMD by more than 65% compared with that of control mice, which might be a result of higher mitochondrial content and decreased inflammation (Mercken et al., 2014). SRT3025 was found to significantly restore bone mass and structure in OVX mice by inhibiting the expression of sclerostin (Artsi et al., 2014). In addition, drugs targeting SIRT1 may also have the potential to treat other bone disorders. For example, activation of SIRT1 may protect against bone loss caused by the use of glucocorticoids and thiazolidinedione, both of which are associated with impaired osteoblast differentiation and, at least in part, due to the diversion of MSCs toward the adipocyte lineage (Briot & Roux, 2015; Hou et al., 2019; Schwartz, 2008). Similarly, the SIRT1 agonist SRT2104 may help alleviate immobilization‐induced osteoporosis (Mercken et al., 2014), a common indication in the aging population. Finally, modulation of SIRT1 expression may prove effective for combatting Paget's disease, which is the second most common bone disorder after osteoporosis, both of which are associated with defects in bone remodeling (Feng & McDonald, 2011). These findings provide compelling evidence suggesting that SIRT1 may serve as a potential therapeutic target for combating osteoporosis and other bone disorders.

### 6.2 SIRT3

Honokiol is a natural biphenolic compound derived from the bark of magnolia trees that can bind to SIRT3 and increase SIRT3 expression and activity (Pillai et al., 2015, 2017). Honokiol has been reported to block agonist‐induced and pressure overload‐mediated heart failure (Pillai et al., 2015) as well as the associated mitochondrial damage and cell death (Pillai et al., 2017), and was accompanied by upregulation of SIRT3 expression and activity. At the time of writing, there have been no studies exploring the effect of honokiol in osteoporosis models. In addition, the aforementioned protective effect of resveratrol on bone might be mediated, at least in part, by SIRT3 as the protective effects of resveratrol were blunted by deletion of SIRT3 (Chen et al., 2015). Therefore, STACs should be used in combination with sirtuin transgenic mice in future mechanistic studies to better understand the roles of each sirtuin in bone.

### 6.3 SIRT6

In 2016, You and colleagues reported the first synthetic SIRT6 activator through screening of pyrrolo[1,2‐a]quinoxaline derivatives (You et al., 2017). Later, cyanidin was found to be a more potent SIRT6 activator and shown to increase both the expression and activity of SIRT6 (Rahnasto‐Rilla et al., 2018; You et al., 2019). Cyanidin could protect cells against oxidative stress, thus reducing the risk of age‐related diseases (Kumar and Pandey, 2013; Smeriglio et al., 2016). A recent animal study demonstrated that cyanidin treatment protected against bone loss in OVX mice by inhibiting osteoclast formation and bone resorption (Cheng et al., 2018). More studies on cyanidin in other osteoporosis models are needed to test its therapeutic effect on bone loss prevention and potentially lead to clinical trials in the future.

More recently, using an activity‐based screening platform, a variety of compounds were found to be able to stimulate SIRT6‐mediated deacetylation against the H3 acetyl‐lysine 9 peptide. It is interesting to note that the majority of these activators consist of a terminal negative charge and linear aliphatic chain. Using further simulation analysis, the same group developed a more potent SIRT6 activator, CL5D (Klein et al., 2020). All these findings provide an important foundation for understanding the physiological function of SIRT6 in vivo and to investigate its potential in osteoporosis treatment.

### 6.4 NAD^+^

NAD^+^ is an essential sirtuin substrate, and the abundance of NAD^+^ regulates the enzymatic activity of sirtuins (Imai & Guarente, 2014; Sauve et al., 2006). Restoration of NAD^+^ improved both the life span and health span in animal models of the Werner syndrome, which is accompanied by severe osteoporosis (Fang et al., 2019; Oshima et al., 2017). NAD^+^ boosters serve as another class of STACs that restore NAD^+^ levels and potentially activate all seven sirtuins (Bonkowski & Sinclair, 2016). Nicotinamide mononucleotide (NMN), a key NAD^+^ intermediate, has been shown to enhance NAD^+^ biosynthesis and ameliorate various pathologies in mouse models (Imai & Guarente, 2014; Mills et al., 2016). Mills et al. (2016) showed that NMN could provide modest protection against osteoporosis in mice. Nicotinamide riboside (NR), another natural precursor of NAD^+^, has been shown to extend the life span in both wild‐type mice (Zhang, Ryu, et al., 2016) and progeria animal models (Fang et al., 2014, 2016). In addition, NR also prevents high‐fat diet‐induced glucose dysregulation (Cantó et al., 2012), noise‐induced hearing loss (Brown et al., 2014), cardiac injury (Xu, Barrientos, et al., 2015), and stem cell‐niche depletion (Zhang, Ryu, et al., 2016). Unfortunately, bone phenotypes were not included in the above reports. Future studies are warranted to investigate the effect of NR on bone mass in animal models of osteoporosis. Another question to be answered is whether NAD^+^ precursors exert their protective effects by activating all or some sirtuins.

## 7 IMPLICATIONS FROM CLINICAL STUDIES

Despite the encouraging evidence from preclinical researches, the clinical use of sirtuin activators for the treatment of osteoporosis is not fully supported by sufficient clinical evidence. Nevertheless, several clinical studies have pointed out the link between sirtuins and osteoporosis. For example, women with osteoporotic hip fracture had lower SIRT1 expression at the femoral neck (El‐Haj et al., 2016). Single nucleotide polymorphisms (SNPs) in SIRT1 (associated with lower SIRT1 expression) were also found to be associated with bisphosphonate‐induced osteonecrosis in an exome‐wide association analysis (Yang et al., 2018). These findings, together with the aforementioned preclinical studies, provided early evidence suggesting that SIRT1 might serve as a potential therapeutic target for the treatment of bone disorders. However, there are still outstanding questions to be answered. For example, whether osteoporosis is triggered by dysregulated sirtuin activity or whether sirtuin dysfunction contributes to the progression of osteoporosis remains to be revealed. Other issues, including the effect of sirtuin activation on fracture risk and the healing of osteoporotic fracture, are important clinical questions to be adequately addressed.

Currently, more than 100 ongoing clinical trials are evaluating the safety and physiological activity of STACs for treating human diseases such as cardiovascular diseases, type 2 diabetes, and inflammation (Baksi et al., 2014; Bonkowski & Sinclair, 2016; Figure 4b). Some of these studies have shown the benefits of sirtuins on the cardiovascular system and metabolic diseases, while others reported little or no effects. Resveratrol is the most studied STAC in clinical trials, with NR playing a promising role (Figure 4c). To the best of our knowledge, none of these STACs have been successfully translated into clinical practice for various reasons (those under clinical trial are not accounted for). However, resveratrol, due to its low bioavailability and potency, has demonstrated variable efficacy in humans (Tomé‐Carneiro et al., 2013). Synthetic STACs, such as SRT1720, failed to enter clinical trials due to limited target specificity (Dai et al., 2018; Huber et al., 2010; Nguyen et al., 2013). On the other hand, SRT2104, which mimics the effects of caloric restriction and extends the life span of male mice (Mercken et al., 2014), has entered phase II trials with few side effects (Hoffmann et al., 2013; Venkatasubramanian et al., 2013). SRT3025 was tested in healthy male volunteers for the treatment of metabolic diseases but the trial was stopped by GlaxoSmithKline (GSK) due to unknown reasons (ClinicalTrials.gov Identifier: NCT01340911). Although NR administration increased blood NAD^+^ levels and was well tolerated, it has not resulted in a striking improvement in any diseases so far (Dollerup et al., 2018; Dollerup et al., 2018; Katsyuba et al., 2020; Martens et al., 2018). Oral administration of NMN was reported to be safe and feasible in healthy people. Furthermore, the trend toward decreased blood glucose levels in healthy participants after NMN treatment is a positive sign (Irie et al., 2020). These findings suggest that challenges and barriers still exist in the translation of STACs to clinical practice. Questions to be answered include optimal dosage, duration of treatment, and long‐term side effects. Therefore, future clinical studies need to be designed with good rationale to determine the dosage and administration duration for specific diseases (Gilmour et al., 2020; Lautrup et al., 2019).

Despite the abovementioned preclinical and clinical evidence for the effects of STACs, clinical trials, including osteoporosis as indication, are very limited. A randomized placebo‐controlled trial showed that resveratrol treatment for 16 weeks significantly increased bone mass in elderly men with obesity (ClinicalTrials.gov Identifier: NCT01412645; Ornstrup et al., 2014). A more recent randomized controlled trial also demonstrated that regular resveratrol supplementation improved BMD in postmenopausal women (Wong et al., 2020). Further clinical trials to examine the effects of STACs alone or in combination with existing anti‐osteoporosis drugs on the treatment of osteoporosis are of clinical interest.

## 8 SUMMARY AND FUTURE PERSPECTIVES

This extensive updated review reveals the important roles of sirtuins in diverse bone disorders. As sirtuins exert various biological effects on bone cells, they and their related underlying mechanisms are promising novel targets for the development of anti‐osteoporotic therapies. Based on the current evidence, SIRT1 appears to be the most promising therapeutic target for the prevention of bone loss as it counteracts imbalanced bone remodeling through its actions on MSCs, osteoblasts, osteoclasts, and possibly hormone signaling pathways. In addition, the cumulative preclinical evidence of the anti‐osteoporotic effects of SIRT1 activity enhancement has laid the foundation for many planned or ongoing clinical trials. Despite significant progress over the past few years, many research questions have yet to be answered. For example, it is important to know whether there is any discrepancy in terms of sirtuin activity with respect to sex and chronological age. Moreover, the biological functions of less studied sirtuins such as SIRT2, SIRT4, and SIRT5 in physiological and pathological conditions have not been adequately investigated. More in‐depth mechanistic studies are expected to provide a better understanding of the effect of sirtuins on individual bone cell type by using tissue‐specific deletion and transgenic animal models. From a clinical perspective, more specific and selective sirtuin activators with clearer pharmacokinetic and pharmacodynamic properties are needed to obtain more consistent and conclusive outcomes.

## ACKNOWLEDGEMENTS

This work was supported by the General Research Fund, University Grants Committee, HKSAR (Ref No. 14163517 and 14120818 to WYW Lee), Health and Medical Research Fund, The Food and Health Bureau, The Government of the Hong Kong Special Adminstrative Region (Ref No 06170546 to WYW Lee), and Start‐up Fund, The Chinese University of Hong Kong, HKSAR (Ref No 4930991 to WYW Lee).

## CONFLICT OF INTEREST

The authors declare that they have no competing interests.

## AUTHORS’ CONTRIBUTIONS

QQL and YWL involved in concept and writing of the manuscript; QQL made the tables and created the figures; YWL, CYC, and QJ revised the manuscript; and QQL, CYC, QJ, and YWL performed analysis of the literature and critical discussion.
